# Association between the COVID-19 pandemic and pertussis derived from multiple nationwide data sources, France, 2013 to 2020

**DOI:** 10.2807/1560-7917.ES.2022.27.25.2100933

**Published:** 2022-06-23

**Authors:** Soraya Matczak, Corinne Levy, Camille Fortas, Jérémie F Cohen, Stéphane Béchet, Fatima Aït El Belghiti, Sophie Guillot, Sabine Trombert-Paolantoni, Véronique Jacomo, Yann Savitch, Juliette Paireau, Sylvain Brisse, Nicole Guiso, Daniel Lévy-Bruhl, Robert Cohen, Julie Toubiana

**Affiliations:** 1 Institut Pasteur, Université Paris Cité, Biodiversity and Epidemiology of Bacterial Pathogens, Paris, France; 2 Department of General Pediatrics and Pediatric Infectious Diseases, Hôpital Necker–Enfants Malades, APHP, Université Paris Cité, Paris, France; 3 Université Paris Est, IMRB-GRC GEMINI, Créteil, France; 4 ACTIV, Association Clinique et Thérapeutique Infantile du Val-de-Marne, Créteil, France; 5 GPIP, Groupe de Pathologie Infectieuse Pédiatrique, Paris, France; 6 Clinical Research Center (CRC), Centre Hospitalier Intercommunal de Créteil, Créteil, France; 7 AFPA, Association Française de Pédiatrie Ambulatoire, Saint-Germain-en-Laye, France; 8 Santé publique France, French National Public Health Agency, Saint-Maurice, France; 9 Obstetrical, Perinatal and Pediatric Epidemiology Research Team, Center of Research in Epidemiology and Statistics, INSERM UMR 1153, Université Paris Cité, Paris, France; 10 National Reference Center for Whooping Cough and other Bordetella infections, Institut Pasteur, Paris, France; 11 Laboratoire CERBA, Saint Ouen l’Aumône, France; 12 Laboratoire Eurofins Biomnis, Lyon, France; 13 Institut Pasteur, Université Paris Cité, CNRS UMR2000, Mathematical Modelling of Infectious Diseases Unit, Paris, France; 14 Institut Pasteur, Paris, France

**Keywords:** Pertussis, epidemiology, COVID-19, lockdown

## Abstract

**Background:**

Interventions to mitigate the COVID-19 pandemic may impact other respiratory diseases.

**Aims:**

We aimed to study the course of pertussis in France over an 8-year period including the beginning of the COVID-19 pandemic and its association with COVID-19 mitigation strategies, using multiple nationwide data sources and regression models.

**Methods:**

We analysed the number of French pertussis cases between 2013 and 2020, using PCR test results from nationwide outpatient laboratories (Source 1) and a network of the paediatric wards from 41 hospitals (Source 2). We also used reports of a national primary care paediatric network (Source 3). We conducted a quasi-experimental interrupted time series analysis, relying on negative binomial regression models. The models accounted for seasonality, long-term cycles and secular trend, and included a binary variable for the first national lockdown (start 16 March 2020).

**Results:**

We identified 19,039 pertussis cases from these data sources. Pertussis cases decreased significantly following the implementation of mitigation measures, with adjusted incidence rate ratios of 0.10 (95% CI: 0.04–0.26) and 0.22 (95% CI: 0.07–0.66) for Source 1 and Source 2, respectively. The association was confirmed in Source 3 with a median of, respectively, one (IQR: 0–2) and 0 cases (IQR: 0–0) per month before and after lockdown (p = 0.0048).

**Conclusions:**

The strong reduction in outpatient and hospitalised pertussis cases suggests an impact of COVID-19 mitigation measures on pertussis epidemiology. Pertussis vaccination recommendations should be followed carefully, and disease monitoring should be continued to detect any resurgence after relaxation of mitigation measures.

## Introduction

Whooping cough or pertussis is a highly contagious respiratory disease transmitted from human to human via aerosolised respiratory droplets and is mainly caused by *Bordetella pertussis*. A resurgence of pertussis has been observed in many countries during the last decade despite widespread vaccine implementation [1]. Most pertussis morbidity and mortality are due to severe clinical forms in young infants that usually require admission to intensive care units. In 2014, the World Health Organization (WHO) estimated pertussis as the cause of 160,700 deaths in children younger than 5 years [2]. In Europe, the European Centre for Disease Prevention and Control (ECDC) reported an increase in pertussis cases between 2014 and 2016, followed by a slight decrease; there were 35,627 cases in 2018 [3].

Since December 2019, the world has been facing another infectious respiratory disease, the coronavirus disease (COVID-19) pandemic caused by severe acute respiratory syndrome coronavirus 2 (SARS-CoV-2) [4]. In France, a lockdown was ordered at the beginning of the first wave of the COVID-19 pandemic on 16 March 2020. Measures included school closures, workplace physical distancing and remote work, banning mass gatherings, travel restrictions and closure of public places; mandatory face covering was implemented on 11 May 2020 [5]. During the following months, the French government successively introduced several bundles of COVID-19 mitigation measures based upon the dynamics of SARS-CoV-2 infection. The French population initially faced difficulties of access to clinical and biological diagnosis and to public health services, with a subsequent delay of general childhood vaccinations despite the mandatory infant vaccinations since 2018 and booster vaccination guideline [6]. We hypothesised that such measures and their consequences might have impacted pertussis epidemiology as suggested for other transmissible airborne infectious diseases [7].

Thanks to a well-established surveillance system for pertussis cases through networks of outpatient laboratories and participating hospitals, and the French ambulatory surveillance for outpatient paediatric cases, we assessed the course of pertussis epidemiology in France between 1 January 2013 and 31 December 2020, and its potential association with COVID-19 mitigation strategies.

## Methods

### Data sources and case definitions

We performed a retrospective analysis using three nationwide data sources from pertussis surveillance systems in France from 2013 to 2020 (see Supplementary Figure S1 for the geographical distribution of these surveillance sites). Those include general population surveillance through two nationwide outpatient laboratories (Cerba, Laboratory 1 and Biomnis, Laboratory 2), which carry out more than 90% of the ambulatory testing for pertussis in mainland France (Source 1) [8], and the monitoring of severe paediatric cases through a nationwide network of 41 participating hospitals (Renacoq network, covering ca 30% of hospitalised paediatric pertussis cases) collecting hospitalised pertussis cases under the age of 1 year (Source 2) [9]. Santé publique France (SpF) and the French National Reference Centre (NRC) for *Bordetella* infections collect data from Source 1 every month, and twice a year from Source 2. For these two sources, a pertussis case was defined as a person with a positive result in a PCR targeting IS*481* (simplex PCR) from nasopharyngeal swabs or aspirates. The quantitative PCR methods used for pertussis surveillance were quality-assessed by the French NRC for *Bordetella* infections [10].

We also included data from the French ambulatory surveillance of whooping cough for outpatient paediatric cases. Implemented in 2002 by the Association Clinique et Thérapeutique Infantile du Val de Marne (ACTIV) and the French NRC for *Bordetella* infections, this surveillance (primary care network pertussis surveillance, Source 3) does not aim to estimate the exhaustive number of pertussis cases but the duration of immunity conferred by pertussis vaccines in children in France [11]. From 2013 to 2020, 76 paediatricians from the ACTIV network throughout France reported patients with suspected pertussis (cough illness). All suspected patients were invited for biological confirmation of pertussis. Cases were defined as either ‘confirmed’ (i.e. positive in culture and/or PCR) or ‘epidemiological’ (i.e. acute cough lasting at least 14 days in a child or adult who was in contact with a confirmed case) [11].

Strengthening the Reporting of Observational studies in Epidemiology (STROBE) guidelines were followed to report the study.

### Statistical analysis

We conducted a quasi-experimental interrupted time series analysis relying on negative binomial regression models [12], with a time unit of 1 month, to analyse the changes in the incidence of positive *Bordetella* PCRs over time. All positive PCR results were included in the models. The models accounted for seasonality (using pairs of sine/cosine terms), secular linear trend, a binary variable to define periods before and after the first lockdown (this variable was coded 0 before 1 April 2020 and 1 thereafter; the actual lockdown started on 16 March 2020; Supplementary Figure S2 shows the timeline of implemented measures) and overdispersion of data. The models also included a dummy variable (with 36-month periods) to adjust for long-term cycles commonly observed in pertussis epidemiology [9]. In such models, an incidence rate ratio (IRR) greater than 1 indicates that the corresponding variable is associated with an increase in pertussis incidence.

Firstly, we carried out the analysis on our most comprehensive data source (Source 1) overall, and then stratified across three pre-specified age groups: pre-school children (0–5 years), primary and secondary school children (6–17 years) and adults (≥ 18 years). We further performed the following sensitivity analyses to assess the robustness of our findings: (i) fitting the data from Laboratory 1 and Laboratory 2 separately, (ii) including the negative samples when available (i.e. modelling the proportion of positive PCR results) and (iii) using a different modelling strategy relying on segmented linear regression with autoregressive errors to account for autocorrelation in the data [12]. Secondly, the analysis was carried out on data from Source 2, relying on the same negative binomial regression model used for Source 1. Thirdly, we did not include the data from Source 3 in the time series modelling because they were collected to evaluate the duration of vaccine-induced immunity and did not fulfil model requirements; instead, we carried out an analysis comparing the number of pertussis cases per month (all laboratory- or epidemiologically confirmed) before and after the lockdown. Differences between groups were assessed by the Mann–Whitney *U* test or the Student's *t* test, when appropriate. We used Stata/SE 15.0 (StataCorp LP, College Station, TX, United States) for all analyses.

## Results

### Outpatient laboratory pertussis surveillance (Source 1)

Over the study period, 17,912 pertussis cases were reported from the two private laboratories (Source 1). The median age of patients with a positive PCR was 20.0 years (interquartile range (IQR): 6.5–46.2), and the distribution among age groups was 23% 0–5 year-olds, 24% 6–17 year-olds and 53% ≥ 18 year-olds. A subset of cases aged 2–20 years between 2013 and 2019 has already been analysed elsewhere for other purposes [8]. Panel A of the Figure shows the evolution of overall pertussis cases for the 96-month study period. Before the lockdown (before 1 April 2020), the average number of cases per month was 204 (standard deviation (SD): 119), with a seasonal pattern (i.e. highest incidence in July months, mean = 315; SD: 180). Long-term epidemic cycles were observed: pertussis incidence was higher in 2013 and between 2017 and 2019, compared with the period 2014 to 2016. After the first lockdown, corresponding to the beginning of a sequence of several mitigation measures (please refer to Supplementary Figure S2 for the dates of implementation), the average number of cases was much smaller, reaching 14 cases per month (SD: 18; p < 0.001).

**Figure:**
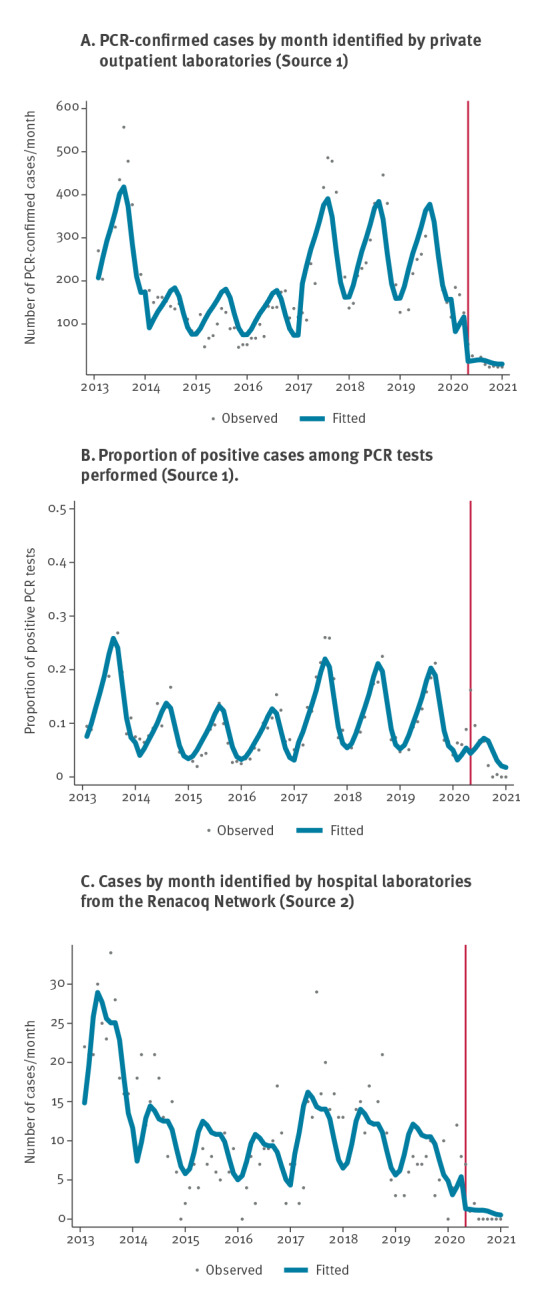
Association between COVID-19 pandemic and pertussis: time series analysis, France, 2013–2020 (n = 18,904)

Fitted values (bold line) as predicted by the negative binomial regression model. The red vertical line indicates the beginning of the first lockdown.

Through time series modelling, we observed a 89.8% decrease (95% confidence Interval (CI): 74.4–96.0) in pertussis cases after the lockdown, corresponding to an adjusted IRR (aIRR) of 0.10 (95% CI: 0.04–0.26) (Table 1). This significant decrease was confirmed in all age groups, with a sharper decrease in the 6–17 year-olds (−92.7%; 95% CI: −97.3 to −79.9) and the ≥ 18 year-olds (−93.6%; 95% CI: −97.5 to −83.5), as compared with the age group 0–5-years (−78.3%; 95% CI: −91.6 to −43.6 ).

**Table 1:** Association between COVID-19 pandemic and pertussis: interrupted time series analysis, France, 2013–2020 (n = 18,904)

Outcome measures	Negative binomial modelling		p value	Segmented linear regression		p value
	Adjusted IRR^a^	95% CI		Change in level^b^	95% CI	
Source 1 – Outpatient laboratories						
Overall number of positive PCRs	0.10	0.04 to 0.26	< 0.001	−242.2	−348.3 to −136.2	< 0.001
**Number of positive PCRs, by age**						
0–5 years	0.22	0.08 to 0.56	0.002	−45.9	−83.2 to −8.5	0.017
6–17 years	0.07	0.03 to 0.20	< 0.001	−61.2	−84.5 to −37.9	< 0.001
≥ 18 years	0.06	0.03 to 0.17	< 0.001	−133.6	−184.2 to −83.1	< 0.001
**Number of positive PCRs, by Laboratory**						
Laboratory 1	0.09	0.04 to 0.24	< 0.001	−103.5	−152.9 to −54.1	< 0.001
Laboratory 2	0.11	0.04 to 0.27	< 0.001	−138.7	−197.3 to −80.2	< 0.001
**Proportion of positive PCRs^c^**	0.67	0.02 to 27.74	0.832	−0.04	−0.13 to −0.04	0.279
Source 2 – Hospital laboratories (Renacoq network)						
Number of positive PCRs in age group < 1 year	0.22	0.07 to 0.66	0.007	Not applicable		

CI: confidence interval; COVID-19: coronavirus disease; IRR: incidence rate ratio.

^a^ The estimated IRR was adjusted for long-term cycles, seasonality (using pairs of sine/cosine terms) and secular trend.

^b^ In contrast to the IRR that represents a relative change, the estimated change in level represents the absolute immediate change in the mean level of the outcome after the COVID-19 lockdown. The estimated change was adjusted for long-term cycles, seasonality (adjustment by calendar month), year and secular trend.

^c^ Proportion calculated using Laboratory 1 data only for 2013–2015, and then data from Laboratory 1 and Laboratory 2 for 2016–2020, because of missing data on negative test results for Laboratory 2 for 2013–2015.

In a sensitivity analysis, our findings did not differ when data from Laboratory 1 and Laboratory 2 were fitted separately (Table 1). When including the negative samples, we observed a non-significant decrease in the proportion of positive PCR results (aIRR = 0.67; 95% CI: 0.02–27.74; p = 0.832) (Figure, panel B, Table 1). Whereas this proportion was 13.4% (SD: 7.6) before lockdown, it fell to 8.3% (SD: 5.2) during the next 5 months and to 0.1% (SD: 0.2) in the final 4 months of 2020 (September to December 2020) despite a relatively stable number of tests during the post-lockdown period (mean = 334 tests per month; SD: 60) (Table 2). Segmented linear regression models yielded results similar to negative binomial modelling, with a significant drp in the number of positive PCR results across all age groups (Table 1).

**Table 2:** Annual number of pertussis cases diagnosed across data sources, France, 2013–2020 (n = 19,039)

Pertussis cases	2013	2014	2015	2016	2017	2018	2019	2020
Source 1 – *IS*481 PCR tests from outpatient laboratories								
**Laboratory 1**								
Positive	2,266	962	450	655	1,473	1,369	1,095	206
Negative	13,139	11,216	8,614	9,112	10,355	12,623	11,336	4,152
Total number of tests	15,405	12,178	9,064	9,767	11,828	13,992	12,431	4,358
**Laboratory 2**								
Positive	1,601	666	552	784	1,881	1,864	1,688	400
Negative	N/A	N/A	N/A	9,372	11,296	13,069	13,819	5,366
Total number of tests	1,601	666	552	10,156	13,177	14,933	15,507	5,766
Source 2 – Hospital laboratories (Renacoq network)								
Number of positive tests	266	149	81	86	162	141	73	34
Source 3 – Outpatient paediatric network (ACTIV)								
Total clinically suspected pertussis	48	27	16	15	37	32	38	7
Confirmed cases	14	13	4	5	14	10	14	2
Epidemiological cases	7	4	4	4	13	6	17	4

N/A: not available.

### Hospital-based pertussis surveillance (Source 2)

Over the study period, 992 positive PCR tests were collected through the Renacoq hospital network (Source 2). The distribution among age groups was 61% 0–2 month-olds, 25% 3–5 month-olds and 14% 6–11 month-olds. We also observed a seasonal pattern (Figure, panel C). Before lockdown, the average number of pertussis cases per month was 11.3 (SD: 7.3). After the lockdown, a sharp decrease in the number of hospitalised pertussis cases was confirmed, with an average case number of 1.1 (SD: 2.3; p < 0.001). In time series modelling, there was a significant association between COVID-19 mitigation measures and pertussis cases, with a reduction of 78.4% (95% CI: 34.4–92.9; aIRR 0.22; 95% CI: 0.07–0.66; p = 0.007) (Figure, panel C and Tables 1 and 2). Between July 2020 and December 2020, no case of pertussis was notified.

### Primary care network pertussis surveillance (Source 3)

From 2013 to 2020, the paediatric outpatient network (Source 3) reported 135 cases of pertussis. Confirmed and epidemiological cases accounted for 76 (56.3%) and 59 (43.7%) cases, respectively (Table 2). Among the 59 epidemiological cases, 25 were adults (family members) and 34 were children (siblings, family members, daycare centre or school). The mean age was 7.1 years (SD: 4.2) for confirmed cases and 22.4 years (SD: 20.1) for epidemiological cases. Before the lockdown, the median number of cases per month was one (IQR: 0–2). After the lockdown, no case of pertussis was identified in Source 3 (median = 0; IQ:R 0–0; p = 0.0048, Mann–Whitney *U* test).

## Discussion

In this 8-year population-based study, we described the dynamics of pertussis epidemiology in France before and after the implementation of the first COVID-19 mitigation measures (16 March 2020, school closure and national lockdown; for all measures by date, see Supplementary Figure S2) [5]. All our data sources highlighted a long-term cyclical pattern commonly observed in pertussis [9], with two high-incidence periods: the year 2013, corresponding to the epidemic from 2011 to 2013 observed in many countries [8,13], and the years 2017 to 2019. Therefore, a decrease in pertussis incidence was to be expected in 2020, such as for the period 2014 to 2016. However, our interrupted time series analysis, accounting for this 36-month pertussis cycle and seasonality, showed an even sharper decrease in pertussis positive PCR tests, with evidence of its association with anti-COVID-19 measures. This drop was particularly marked, with almost no pertussis cases seen after the lockdown, a level never observed since the beginning of the French pertussis surveillance in 1996 [8].

The decrease in pertussis cases following the mitigation measures occurred in all age groups. However, it was less pronounced in the 0–5-year group tested by the two private laboratories. One explanation could be that the youngest children were more likely to visit their paediatrician despite lockdown measures. Another explanation could be that transmission might still have been active in this age group during the first months of 2020. We did not find a significant association between the proportion of positive PCR tests and the mitigation measures against SARS-CoV-2. Our data show that the overall number of PCR tests decreased right after the lockdown, while pertussis might still have circulated in households. However, despite a steady number of PCR tests performed during the whole post-lockdown period, the number of positive cases reached almost zero in the last 4 months of 2020; of note, access to clinical and biological diagnosis went back to normal during that period. All these findings suggest a significant decrease of pertussis circulation in France.

Previous studies have revealed a major impact of COVID-19 pandemic mitigation measures on transmissible diseases, such as, in the paediatric population, gastroenteritis, common cold, bronchiolitis and acute otitis media, but also measles [7,14]. However, a resurgence of some other common airborne infectious diseases has been observed in the setting of relaxed physical distancing recommendations, such as the delayed inter-seasonal respiratory syncytial virus (RSV) epidemic in France and in other parts of the world [15-17]. For now (December 2021), we have not observed a pertussis resurgence despite the end of the first lockdown and school fully reopening in France. Our data suggest that, unlike for RSV, slight relaxation of public health measures (with maintained physical distancing and mask wearing) had no impact on pertussis dynamics; the same was the case for the influenza virus for which no outbreak was noted in France and worldwide in 2020 [18-20]. We identified six studies, including two European studies from Italy and Sweden, describing the trends of several infectious diseases, including pertussis, before and after implementing their respective COVID-19 mitigation measures [21-24]. Although the power of these studies was much lower compared with ours (lower duration of surveillance and description of a maximum of ca 1,500 cases, vs 19,039 cases in our study), they found a decrease in pertussis cases following the implementation of mitigation measures, with reductions ranging from 28% to 87% [21-26].

Even if the incidence of pertussis has strongly decreased since the introduction of booster vaccines, *B. pertussis* is still circulating in France, especially in older children, adults and elderly people, as we have previously shown [8,9,11]. This may be due to non-optimal vaccination coverage in these populations [27] and waning of immunity with the acellular pertussis vaccine [8,28]. The limited circulation of *B. pertussis* in the community may be a clear positive collateral effect of the measures imposed by the COVID-19 pandemic. However, this reduced bacterial circulation may decrease the herd immunity, with the subsequent risk of a larger epidemic in the coming years, after relaxation of all mitigation measures [29]. Most concerns are related to vaccine uptake; during the lockdown, paediatricians involved in data Source 3 declared that they followed vaccinations guidelines [30]. However, as in many countries, delays in vaccination have been noted in the general population in France since the beginning of the pandemic; they mostly concerned booster vaccinations for children and adults [6,31]. Such delays can be dangerous for infants under 6 months of age who are not or partially vaccinated, notably if there is no catch-up before a potential resurgence of pertussis.

Our study has limitations. Firstly, measurement bias is present in all data sources, as Sources 1 and 2 may have missed cases diagnosed by serology (sometimes prescribed even if not recommended) or cases diagnosed on clinical grounds, especially during the first lockdown (from mid-March to May 2020) when transportation was limited, office-based physicians could not be reached and private laboratories were overwhelmed by the implementation of large-scale SARS-CoV-2 testing. Secondly, we cannot determine whether the decrease in pertussis cases observed here was due to decreased pertussis circulation or reduced testing. However, the similar decrease observed in hospitalised cases in the youngest population does not favour the latter hypothesis, and access retrictions to outpatient testing were mostly limited to the first lockdown period. Thirdly, we may have lacked statistical power to detect a significant decrease in the proportion of positive cases, as the post-lockdown period was relatively short compared with the pre-lockdown period (9 months vs 87 months) and negative PCR tests results were not available from data Source 2. Finally, adherance to mitigation measures was not measured at the patient level, we cannot confirm that the observed drop in pertussis cases was a direct consequence of these measures.

## Conclusion

This national-level study shows a strong association between the COVID-19 pandemic and the trend in pertussis incidence in France, with an unprecedented drop in pertussis cases. Pertussis should be closely monitored to detect any resurgence in the community when physical distancing restrictions are relaxed, as well as compliance with vaccination of infants, children and adults.

## Supplementary Data

378000 Supplement
