# Supplementary material for: Association between the COVID-19 pandemic and pertussis derived from multiple nationwide data sources, France, 2013 to 2020
Source: Euro Surveill. 2022 Jun 23;27(25):2100933. doi: 10.2807/1560-7917.ES.2022.27.25.2100933 (PMC9229195; doi:10.2807/1560-7917.ES.2022.27.25.2100933)
Supplement: Supplement [file 21-00933_TOUBIANA_Supplement.pdf]

Supplementary files

Association between the COVID-19 pandemic and pertussis derived from multiple nationwide data sources, France, 2013 to 2020

This supplementary material is hosted by Eurosurveillance as supporting information alongside the article "*Association between the COVID-19 pandemic and pertussis derived from multiple nationwide data sources, France, 2013 to 2020*" on behalf of the authors who remain responsible for the accuracy and appropriateness of the content. The same standards for ethics, copyright, attributions and permissions as for the article apply. Supplements are not edited by Eurosurveillance and the journal is not responsible for the maintenance of any links or email addresses provided therein.

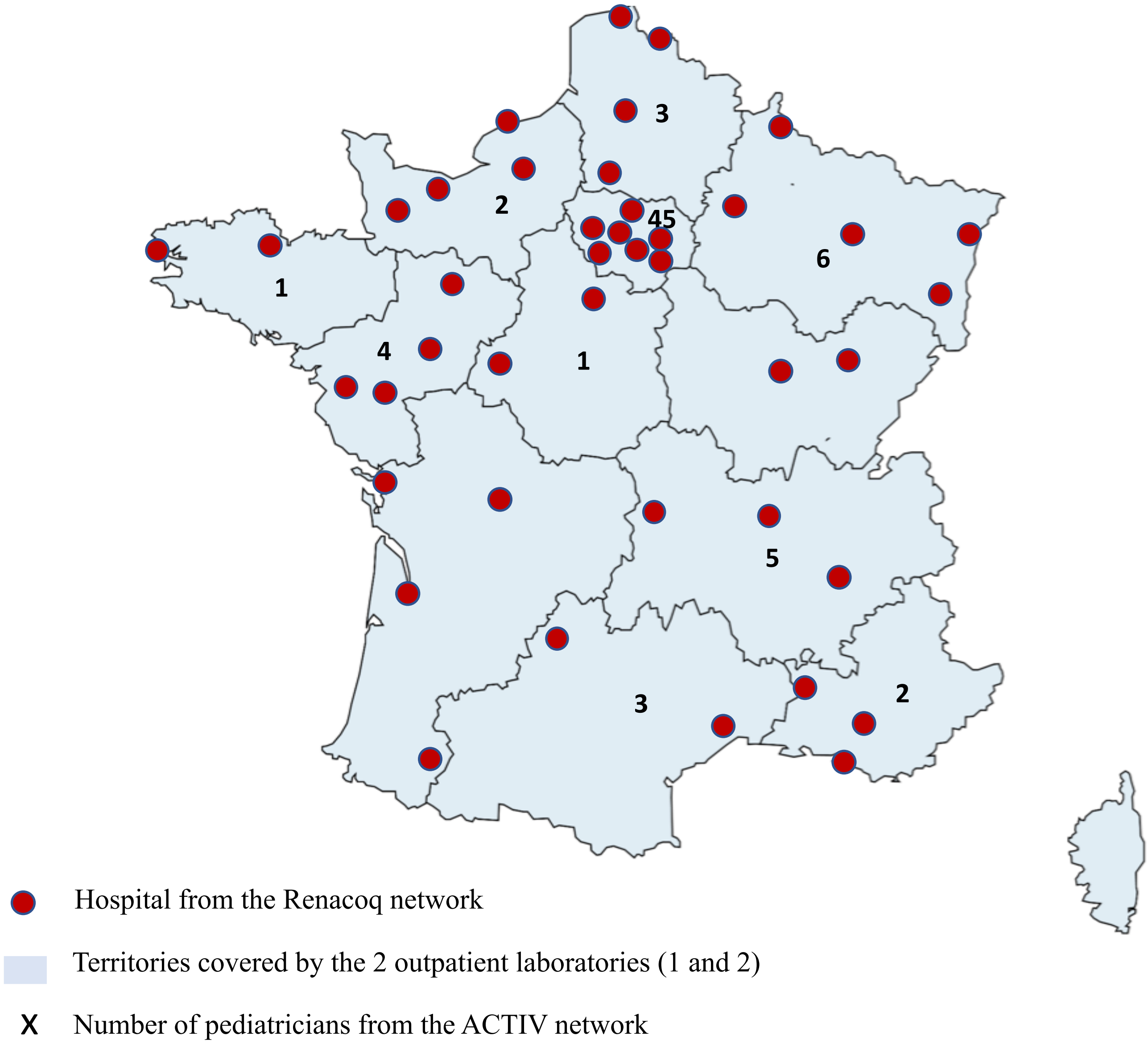

Supplementary Figure 1: Surveillance sites all over France for Renacoq and private laboratories (CERBA and Biomnis)

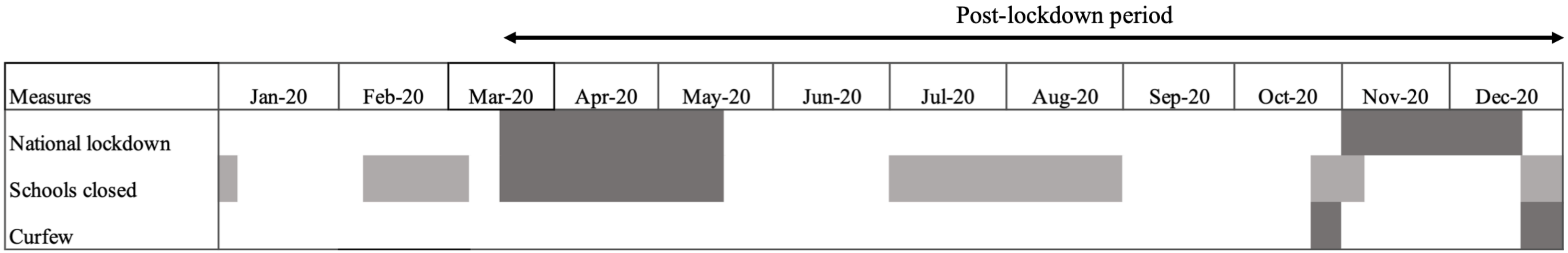

Supplementary Figure 2. French COVID-19 mitigation measures in 2020. Mitigation measures are represented in dark grey, periods when schools were closed because of holidays are represented in light grey
